# Factors influencing willingness to participate in new drug trial studies: a study among parents whose children were recruited into these trials in northern Ghana

**DOI:** 10.1186/s13104-016-1951-4

**Published:** 2016-03-03

**Authors:** James Akazili, Samuel Chatio, Fabian Sebastian Achana, Abraham Oduro, Edmund W. Kanmiki, Frank Baiden

**Affiliations:** Navrongo Health Research Centre, P.O Box 114, Navrongo, Ghana; Ensign College of Public Health, ER, Kpong, Ghana

**Keywords:** Willingness to participate, New drug trials, Kassena-Nankana districts, Northern Ghana

## Abstract

**Background:**

During the last decade, the number of clinical trials conducted in sub-Saharan Africa has increased significantly which has helped to address priority health problems in the region. Navrongo health research centre since it was established in 1989, has conducted several trial studies including rectal artesunate trial in the Kassena-Nankana districts. However, there is little evidence-based for assessing the impact of new drug trials. This study explored factors that motivate parents to allow their children to participate in new drug trials in northern Ghana.

**Method:**

The study used both quantitative and qualitative methods. The participants were randomly selected from among parents whose children were enrolled in a new drug trial conducted in the Kassena-Nankana districts between 2000 and 2003. QSR Nvivo 9 software was used to code the qualitative data into themes before analysis while STATA software Version 11.2© was used to analyze the quantitative data.

**Results:**

The results showed that majority (95.9 %) of the parents were willing to allow their children to be enrolled in future new drug trials. The main factors motivating their willingness to allow their children to be enrolled in these trials were quality of health care services offered to trial participants (92.9 %), detail medical examination (90.8 %), promptness of care provided (94.4 %) and quality of drugs (91.9 %). Other factors mentioned included disease prevention (99.5 %) and improved living standard (96.1 %). Parents reported that the conduct of these trials had reduced the frequency of disease occurrences in the communities because of the quality of health care services provided to the children recruited into these trial studies.

**Conclusion:**

Though the implementation of clinical trials in the study area is believed to have positive impact on health status of people particularly trial participants, measures should however be taken to address safety and likely side effects of new drugs given to trial participants during these trial studies.

## Background

During the last decade, the number of clinical trials conducted in sub-Saharan Africa has increased significantly. This has led to the creation of new funding mechanisms for clinical research which has helped to address priority health problems in the region [1]. Clinical trials research before the year 2000 was mainly carried out in affluent countries with the sponsorship and funding from pharmaceutical industry. Today trial studies are also carried out in resource-constrained contexts, and they are often sponsored by academic or not-for-profit organizations with some being funded externally. Reliable evidence is essential to improve health care delivery and to support efficient use of limited resources. Randomized clinical trials (RCTs) are the most rigorous method of generating information to help patients, clinicians, policy-makers and funding agencies make informed clinical and health policy decisions. Without this evidence, there is a risk that people could be given treatments that might be harmful to them [2]. Evidence exists that clinical trials are conducted to test the efficacy and safety of new drugs and this allows researchers to come out with new drugs for the treatment of certain diseases in communities [2–4].

It is established that about 62 % of trials participants had no knowledge on the conduct of new drug trials [5]. This could be attributed to poor sensitization and education prior to new drug trial implementation. It might also be blamed on poor consenting before trial participants take a decision to participate in these trials [6–8]. It has also been established that misconceptions about the use of samples affects recruitment and retention of participants into trial studies [9, 10]. There are certain risks that trials participants are exposed to when they take part in these trial studies. Trials participants are not sure of the efficacy and side effects of these new drugs they receive [4, 11]. Other risks such as getting infected with virus, worsening of health status, damage to the liver and kidney have also been mentioned as likely risks associated to new drug trials [3]. It is demonstrated that trial participants felt they were being used as guinea pigs because of the placebo participants received during these trials [2, 3]. The conduct of clinical trials in Sub-Saharan Africa has significantly increased over the years. Therefore, there is the need to examine these challenges more closely and also document the impact of the conduct of these trials to participants and their families.

Navrongo health research centre (NHRC) located in central Navrongo was establishment in 1989 with the mandate of conducting high quality demographic and health research to inform policy. The research centre started with vitamin A supplementation trial (VAST) study which was conducted to assess the impact on childhood morbidity and mortality in northern Ghana [12]. The trial made a huge impact in the study communities and because of that the term VAST has been used synonymously with the research centre by community members. The centre over the years has conducted several other trial studies including rectal artesunate trial in the study area. The artesunate trial was conducted from 2000 to 2004 with a little over 2000 children enrolled into the study. The trial was both community and facility-based, and had over 45 fieldworkers and supervisors living in the communities to recruit cases into the study [13]. However, there is little evidence-based for assessing the impact of these trials on communities where these trials take place especially people who are recruited into these trials. Little evidence also exists in determining the attitude and willingness of people to participate in new drug trial studies. This study was designed to explore factors that motivate parents to allow their children to participate in these trials in the Kassena-Nankana districts of northern Ghana.

## Methods

### Study design

The study used mixed method where both quantitative and qualitative techniques were used for data collection. The quantitative survey presents figures and percentages on research problem whilst qualitative interviews encouraged people to explain in more detail their personal opinions and experiences on the problem under investigation. Both interviews were conducted with parents whose children were recruited into rectal artesunate trial and other trials conducted in the study area. The qualitative component of the study was done first and issues that came up in the data informed the study team on the type of questions to be asked in the survey. This was to enable the study investigators to test the significant of these issues quantitatively.

### Study site

The study was conducted in the Kassena-Nankana east and west districts (KNEWDs) of northern Ghana. The area shares borders with Burkina Faso in the north and covers an area of about 1675 km^2^ and an estimated population of approximately 152,000 under surveillance by the Navrongo health demographic surveillance system (NHDSS) [14] operating under the NHRC. The districts have two distinct seasons, a wet season that runs from May to September and a long dry season from October to April with hardly any rains. There are two main ethnic groups in the area, the Kasenas and the Nankani speaking people.

The districts have five health centers, two clinics, and 27 functional Community-based health planning and services (CHPS) compounds located in various villages with resident community health officers (CHOs) offering doorstep health care services to the people [15, 16]. The main referral hospital (War Memorial Hospital) is located at the capital (Navrongo town) of the KNED.

### Sample size calculation

We aimed to interview 380 randomly-sampled parents whose children were recruited into rectal artesunate trial. This was based on the assumption that we would be able to estimate the acceptability of new drug trials within a margin of error of 5 %, at 95 % confidence level. Given the length of time between the time the trial was conducted and the time of the current survey, we allowed for a 20 % rate of non-responders.

### Sampling technique

The NHRC operates a demographic surveillance system (NDSS) in the Kassena-Nankana east and west districts. The districts have been divided into five zones (north, south, east, west and central) [14]. Each zone is sub divided into clusters/communities and further into compounds. There are about 270 clusters and 17, 855 compounds that have been enumerated and given compound numbers (COMPOUND IDs). Members in each compound are given unique identity numbers (PERMANENT IDs) for easy identification, and information on births, pregnancies and other vital demographic events are collected and updated at every 120 days interval [14]. The interviews were conducted in three zones (north, south and central) in the two districts. A cluster sampling technique was used in this study. Simple random sampling technique was used to select two zones (north and south) and central zone which is located in the central Navrongo was automatically selected.

Simple random sampling technique was used to select from among parents whose children were enrolled in the artesunate trial which was conducted in the districts between 2000 and 2003 (ISRCTN46343627). The trial was randomized-controlled study that assessed the efficacy of using rectal artesunate pre-referral medication. The trail was conducted to assess the effect of the artesunate medication on child mortality in the districts [13]. The selection was done proportionately to the population or number of artesunate trial participants who were recruited into the trial in each of the three zones taking into consideration the total sample size for the study. Twenty percent was used to select additional list in each of the zones to take care of respondents who were likely not to be available at the time the interviews were conducted. Rectal artesunate trial participants were selected because there existed a database of the parents of children enrolled in the study.

Since this trial ended in 2003 and a number of other trials have taken place in the study area that targeted children in the same age band, it is conceivable that the participants sampled in this study may have had experience from other trials. We therefore anticipated that their views on clinical trials as expressed in this study could not be solely attributed to their experience during the index trial. Simple random sampling method was also used to select respondents for the in-depth interviews. Using a list of children who participated in the artesunate trial as the sample frame, potential participants in this study were randomly selected in all the zones and a list generated for the In-depth interviews. The principle of saturation was used where the data collection, processing and analysis were done concurrently until no new information was obtained. A total of 80 in-depth interviews were conducted with parents whose children were recruited into rectal artesunate trial.

### Data collection technique and tools

A structured survey questionnaire was used to interview respondents. The potential respondents selected were followed up by trained data collectors using respondents’ background information such as name of study child, name of mother, compound name, compound ID, cluster ID and name of village. The data collectors visited potential study participants and those who granted consent were interviewed. The interviews were conducted until the sample size of 380 was achieved.

Interview guide was used to conduct the in-depth interviews with parents. Interview moderators were assigned to the various zones and potential participants contacted to establish their availability and willingness to be interviewed. After obtaining consent from study participants, suitable arrangements were made for the interviews to be conducted at home.

### Training of data collectors

Senior high school graduates were recruited and trained for the quantitative data collection whilst first degree graduates with experience in qualitative interview moderation were recruited and trained to conduct the in-depth interviews. The training for both groups lasted for 1 week and covered areas such as the purpose and objectives of the study, quantitative and qualitative data collection techniques, how to obtain informed consent from study participants and translation of the questionnaire into the various local languages used in the study area (Kasem and Nankam). Role-play was done by all the data collectors to help them have a better understanding of how to ask the questions appropriately during the actual data collection. The questionnaire and the guides were both pre-tested which enabled the study team to finalize them for the actual interviews.

Supervision was very key to the successes of the study. Therefore, at each stage of the data collection, the data collectors were supervised by a field supervisor to make sure that the data collection was done accurately. All completed questionnaires were checked by the field supervision to resolve all queries. All questionnaires with problems were given back to the data collectors responsible to go back to the field and resolve such issues before the forms were submitted for data entry.

### Data processing and analysis

The quantitative survey data was double-entered and verified using Epidata 3.1 with built in consistency checks to control data input. Data cleaning by way of identifying outliers and checking for consistencies among variables were carried out by running frequencies and cross tabulations using STATA software Version 11.2©. Descriptive analyses were used to describe socio-demographic characteristics of respondents. The statistical point estimates were computed and presented as percentages for all the background characteristics and views on factors that influence community members’ willingness to participant in clinical trials.

The qualitative interviews were tape recorded and transcribed verbatim and entered into a computer using Microsoft word. A coding list was prepared to guide the data coding and analysis. Data was organized using QSR Nvivo 9 software before analysis. The data was coded twice where the first coding was used to code the data into major themes while the second coding was used to further code the data into more specific sub-themes. The data analysis was going on simultaneously with data collection. This was to make sure that information was collected to cover all the themes. Codebook was developed based on the major themes of the study and transformed into tree notes and free notes. Common themes were highlighted and quotes were selected and used to support themes in the results.

### Ethical considerations

Ethical approval for the study was received from the NHRC institutional review board (IRB). Written informed consent was obtained before the interviews were conducted. The data collectors read and translated the consent forms into the preferred local language of the participants. All participants were informed before the start of the interview about project goals, how long the interview would last, their right to refuse being interviewed, right to withdraw in the process of the interview and about the intended use of the results for scientific publications and also disseminate the findings to stakeholders. To ensure confidentiality, identification numbers were assigned to participants in the qualitative interviews instead of their names.

## Results

### Socio-demographic characteristics of respondents

The results showed that majority of the respondents 88.6 % (349) were females whilst most of the participants 56.4 % (222) fell between the ages of 31 and 44. From the results, 64.5 % (254) of respondents never had formal education while only about 0.5 % (2) went up to Tertiary/higher level. The results showed that more than half of the respondents (60.4 %) were subsistent farmers with only 1 % (4) of them being civil/public servants (Table 1).

**Table 1:** Socio Demographic Characteristics of Respondents

Variables	N = 394 frequency	Percentage
Age of respondent		
18–30	50	12.7
31–44	222	56.4
45+	122	30.9
Sex		
Male	45	11.4
Female	349	88.6
Educational status		
No education	254	64.5
Primary	88	22.3
Middle/JSS	39	9.9
Secondary	11	2.8
Tertiary/higher	2	0.5
Religion		
Traditional	93	23.6
Christian	270	68.5
Muslim	24	6.1
Other	7	1.8
Ethnicity		
Kassem	171	43.4
Nankam	200	50.8
Buli	17	4.3
Other	6	1.5
Marital status		
Never married	5	1.3
Married/living	340	86.3
Together	9	2.3
Devoiced	39	9.9
Windowed	1	0.2
Main occupation		
Subsistence farmer	238	60.4
Trader	141	35.8
Housewife	3	0.8
Civil/public servant	4	1.0
Unemployed	2	0.5
Other	6	1.5

### Parents expectations before allowing their children to take part in these trials

Most of the parents reported that what they expected before allowing their children to be enrolled in these new drug trial studies was achieved. They mentioned good health for their children as the main thing they expected. They were of the view that their participation in these studies made them to get good health care, good drugs and appropriate attention and treatment for their children and that ensured their good health. Others were of the opinion that they wanted their children to be taken care of fully including payment of their hospital bills by trial studies and that necessitated their involvement in these trial studies. Few other parents reported that they wanted to receive health education on how to take proper care of themselves and their children which to them was achieved. The views of parents are exemplified in these quotes below:

R: “Yes I had in mind that VAST was going to pay for all the drugs or treatment at the hospital for me which they did. …so if you are recruited by VAST into their studies, they will pay for all the treatment for you and they will even treat your child to get well and that is why you have to be part of it” (*IDI*-*41* *year old Mother*).

Q: And do you normally get the good health?

R: “of course very well, when my child took part in that study, he has never had that type of sickness since then so because of that I have seen that it is beneficial to me” (*IDI*-*41* *year old Mother*).

### Attitude and trust towards the conduct of new drug trials

The results showed that majority 95.9 % (378) of the parents were more than willing to allow their children to be enrolled in future clinical trials (Table 2).

**Table 2:** Attitude and trust towards the conduct of new drug trials

Variables	N = 394 frequency	Percentage
Wiliness to participate		
Yes	378	95.94
No	5	1.27
Don’t know	11	2.79
Trial drugs test on human beings		
Yes	305	77.4
No	85	21.6
Don’t know	4	1.02
New drug test on child		
Yes	304	77.2
No	88	22.3
Don’t know	2	0.5

Most of the parents 77.4 % (305) were of the opinion that new drugs should be tested on human beings. On the contrary, about 22 % (85) of them reported that new drugs should not be tested on human beings. Interestingly from the results, it is observed that the number of participants who said it is good for new drugs to be tested on human beings is by one less than those who said they would actually give permit for their children to be participated in trial studies. Again, two people who could not tell whether trial drugs should be tested on human beings or not however said they would allow their children to participate in clinical trials (Table 2).

Similar sentiments were expressed in the qualitative interviews. Most of the parents said that they were committed and willing to cooperate and support the conduct of new drug trials. They reported that the artesunate trial and other trial conducted in the past had contributed immensely to improve community trust in the formal health system. Apparently, community members actively monitored the health progress of children recruited into the study. This coupled with positive feedback by mothers and caregivers to family and community members bolstered the image of the trial and by extension the health system among community members. Therefore, the positive impact of past clinical trials including the artesunate trial has made community members to have positive views and attitude towards the conduct of clinical trials in the study area. Participants offered these views about their attitude and trust towards the conduct of clinical trials.

“When you are usually recruited into a study and they give your child drugs, people in the community will always come around and ask, how is the child doing after taking the drug? And because the drug has worked well for you, you will tell them it is good it is improving my child’s health. Because of that work, people now patronize the hospital, when they are sick, they go there knowing very well that that is where they can be cured” (*IDI*-*48* *year old Mother*).

“We trust the VAST workers because they suffer a lot for our sake, taking care of us and our children, is good. May God help them and make their work develop” (*IDI*-*47* *year old Mother*).

### Main factors motivating willingness to participate in trial studies

NHRC since its existence over 25 year ago has carried out quite a number of new drug trial studies in its research communities. The cooperation, support and the involvement of community members in these trial studies has been tremendous. What has motivated parents and other community members’ high level of cooperation, support and trust to always participate in these trial studies over the years is discussed below.

#### Quality of health care services provided to trial participant

The results demonstrated that quality of health care services offered to trial participants greatly influence their trust and willingness to participate in new drug trials. Study participants were made to compare health care services offered to clinical trials participants and those who were not recruited into clinical trial studies when they both met at the health facility. The results showed that 92.9 % (366) respondents said there were differences in the health care services offered to clinical trial patients and routine patients at the health facility level. Majority of the parents in this study reported that trial studies participants received quality health care services as compared to their colleagues who were not recruited into these trials. The results further established that 90.8 % (357) and 94.4 % (371) of parents said that trial patients were given detail medical examination and promptness of care respectively unlike their colleague children who were not part of these trial studies (Table 3).

**Table 3:** Quality of health care services to trial participants

Variables	N = 394 frequency	Percentage
Differences in health care		
Yes	366	92.9
No	20	5.1
Don’t know	8	2.0
Quantity of drugs		
More drugs for participants	358	90.9
More drugs for routine patients	3	0.8
Same drugs for all	20	5.1
Don’t know	13	3.3
Quality of drugs		
Quality drugs for participants	362	91.9
Quality drugs for routine patients	5	1.3
Same quality drugs for all	15	3.8
Don’t know	12	3.1
Promptness of care		
Trials participants	371	94.4
Routine participants	4	1.0
No difference	11	2.8
Don’t know	7	1.9
Detail examination		
Trial participants	357	90.8
Routine participants	4	1.0
No difference	22	5.6
Don’t know	10	2.5
Cost of drugs/medicine		
Less cost for participants	378	96.9
Less cost for routine patients	0	0.0
No difference in cost	7	1.79
Don’t know	5	1.28

Similar views were expressed by parents in the qualitative interviews. They perceived that clinical trials led to improvement of health care services especially those who were recruited into these trial studies compared with routine patients when they both met at the health facility. Most of them reported that clinical trial patients received quicker and faster care and they were not allow to join long queues and as a result spent shorter time at the health facility as compared to routine patients. Staff were more patient towards trial patients, review their medical history and follow-ups monitoring visits were made by trial staff to ensure that study children were very healthy following treatment given them at the health facility. The following expressions reflect views on quality of health care services offered to trial participants.

“When you are referred there by the VAST people they would take good care of you quickly than when you go there alone for routine care. The reason is that I was having a child who was part of a one year study and when the child was sick, I took him there and when I got there the vast workers took the child quickly and sent him to the doctor for treatment. When the study was over and the child was not part of it again, we went to the hospital and we had to go and join the queue before we could see the doctor unlike the first one where they just took the child straight from me to the doctor for examination. So to me, if you are part of a study the care is faster than the routine care” (*IDI*-*39* *year old Mother*).

“During the time my child was a study child, any time he fell sick, the vast workers came and took him away for treatment. If a child is not a study child and falls sick, the mother would put him on her back, walk and by the time she gets to the hospital, there would be a crowd there. Your child is not the only patient and if you do not know anyone there, they will not attend to you, you have to do “*wahala paa*” (suffer a lot) before you may get to see the doctor. But with the coming of the study, the vast workers work for everyone, rich or poor. They only want to save humanity so they pick you there for treatment. It was better when the study was running” (*IDI*-*44* *year old father*).

#### Quantity, quality and cost of medications

Most of the respondents 90.9 % (358) said that clinical trials children received more drugs at the health facility than those whose children were not recruited into these trial studies. Only few respondents 0.76 % (20) thought otherwise. Similarly, most respondents 91.9 % (362) reported that drugs given to clinical trial children were of better quality and more effective than those given during routine care. With regard to the cost of medications, most participants 96.9 % (378) said that drugs given to clinical trial children were less expensive (Table 3).

Findings were affirmed during the qualitative interviews where in-depth explanations were given on the basis of the suspicion that drugs given during clinical trials were of better quality than those given during routine services. Parents pointed a practice in the routine care where health workers poured medicines from containers into bottles for patients and in the process “reduced the “strength” (efficacy) of the medicine”. Suspicions were also raised regarding the possibility of medicines being diluted with lots of water. In contrast, trial patients were mostly served with pre-packed drugs which in the perspective of parents offered high efficacy hence facilitated their willingness to allow their children to take part in these trial studies.

The vast (refers to NHRC) people give us more drugs and quite apart from that their drugs are even of more quality than the hospital drugs. The reason is that at the hospital, they will only give you paracetamole and quinine which may not be what is needed to treat the illness. The way they also do with the bottle medicine is not good. They could take one bottle and share it with two people and when they open it and pour the medicine in a different bottle, the effectiveness of the medicine reduces….The way they delay if it happens that your child sickness is very serious then of course you will go back home with a dead child (*IDI*-*38* *year old Mother*).

If your child is not a study child, you will pay so much money; only two bottles of medicine and the amount of money they will ask you to pay, you will not know what to do. But when they recruited my child into the study, whenever I got there they gave us all the medicines; they gave us medicine to cool down his body when he had high temperature, medicine for diarrhea and vomiting, everything and I did not pay even a pesewa. Those who were not part of the study paid so much without getting that quantity of medicines (*IDI*-*47* *year old Mother*).

#### Disease prevention and good health

The results in the quantitative survey showed that majority of the respondents 99.5 % (392) said that the conduct of clinical trials had tremendously reduced the frequency of disease occurrences in the communities. With regards to malaria prevention, 98.5 % (388) of the parents said that the conduct of clinical trials had led to a drastic reduction in the prevalence of malaria in the study area. In the same way, 93.1 % (367) of them reported that the conduct of clinical trial had led to a reduction in deaths (Table 4).

**Table 4:** Prevention of Diseases, deaths and living standards of trial participants

Variables	N = 394 frequency	Percentage
Reduce frequency of diseases		
Yes	392	99.5
No	2	0.5
Reduce frequency of deaths		
Yes	367	93.1
No	23	5.9
Don’t know	4	1.0
Malaria prevention		
Yes	388	98.5
No	5	1.3
Don’t know	1	0.3
Improve living standards		
Yes	373	96.1
No	14	3.6
Don’t know	1	0.3
How living standard improved		
Refund	54	13.9
Free medical care	97	25.0
Improve child health	225	58.9
Employment	2	0.5
Other	16	1.7

It was observed in the qualitative interviews that these trials had helped to improve health of their children. Parents were of the view that in the past, there used to be many “strange diseases” that killed people especially children in their communities. They held a strong believe that due to the research activities of NHRC, most of these diseases have been eradicated. Most of them believed that it was as a result of clinical trials including the artesunate trial conducted by the NHRC in the past that had resulted in the eradication of diseases such as measles and convulsion in their communities. They explained that the prevalence of certain diseases such as malaria, cerebrospinal meningitis, elephantiasis, cholera and diarrhoea have declined due to good health care services offered to them by health workers who work directly or indirectly in these trial studies. View expressed in the discussions suggested that the conduct of clinical trials have contributed to reduce infant mortality in the study area. Parents expressed their views this way on the issue:

“The benefits are the way they cure the sicknesses. If not for their medicine how was one going to carry his/her child all the way to the hospital, you may get there and it would be too late but for the sake of that drug that they put into the child’s anus before you send the child to hospital is the reason why I am saying that their work is good. Let me compare it with those of us they recruited our children. I must say that the drug is good because it has helped our children to be healthy. We are better than those they did not recruit their children…”(*IDI*-*40* *year old Mother*).

“Before the Artesunate trial, childhood diseases were so many; but with the coming of the trial the diseases have reduced. Only children who are destined to die fall sick these days. At first diseases like “kachuwa” (measles), chicken pox and “weyusunga” (convulsion) were common but the study brought so many medicines for children, so the diseases that used to worry them have reduced” (*IDI*-*34* *year old Mother*).

#### Trials influence on living standards of trial participants

The findings showed that 96.1 % (373) of the respondents said that the conduct of trials had greatly improved their living standards because of their involvement in these studies (Table 4).

Almost all the parents in the qualitative interviews were of the opinion that trial studies had contributed immensely to improving their living standards. They explained that the free medical care offered by these trials, enables them save money which was used to buy food and other household items for the up keep of the family members. It also enables them to save money to educate their children who after completion of their studies got employed in other sectors which have helped to reduce the level of poverty and improved their living standards. In the words of one participant:

“Yes the studies have helped in this regard. You know that ill health is usually what makes people unable to work but once people are healthy, they are able to do their work to better their life” (*IDI*- *trial participant’s parent*).

### Perceptions on exposure to risks during clinical trials

Few of the parents in the qualitative interviews however, highlighted some issues they did not like when their children took part in these trial studies. They held the view that taking of blood samples from sick children was painful and has made the children to cry. They added that children with low hemoglobin, researchers’ still took blood samples from them. A female parent shared her views this way on the issue:

Q: What problems or risks did your child get in the trial study you took part?

R: You always think that, “oh, this child is sick and yet they are taking his/her blood; his blood is not much” but what can you do?….(*IDI*-*39* *year old Mother*).

Adverse effect was also reported by few of the parents in the in-depth interviews. They mentioned burning of triode as a result of the use of new drugs as the main side effect trial participants were likely to experience. A 48 year old parent expressed his views this way on the issue:

“Yes there are risks because you test and the results are positive, you test and the results are negative and so there is a risk there but you cannot actually succeed in life without taking risks. Let’s say that this drug is taken orally, it can burn your triode when you are taking it and the intestines can be destroyed and you can be sorry and these are some of the risks involved” (*IDI with 48* *year old father*).

## Discussion

### Attitude and trust towards the conduct of new drug trials

People in the study districts continue to have so much trust in the conduct of new drug trials. The results showed high level of trust and positive attitude towards the conduct of clinical trials by stakeholders and other community members particularly parents whose children were recruited into the artesunate trial and other trials conducted in the by the NHRC. Most of the parents reported that they are more than willing to be involved in future clinical trials. This is because of the positive impact and the numerous benefits people continue to get from their participation in these trial studies. This was further affirmed by the finding that a significant number of participants were willing to participate in clinical trial studies [2, 17]. People are willingness and ready to participate in trial studies because it would help researchers to come out with new drugs to benefit their families and relatives [3, 4, 18].

Parents’ willingness, attitude and trust towards the conduct of new drug trials in our study have been high. Most of the parents said that they are committed and willing to cooperate and support the conduct of new drug trials. This support and cooperation has been attributed to the initial trials conducted in the communities by NHRC including the artesunate trial which made huge impact on the lives of community members. For instance, the term VAST has become synonymous with the research centre largely owing to the fact that it was the first trial in this district but also because the trial achieved good results and the community members could easily still remember the trial [12].

### Quality of care and medications

Better quality of care given to trials patients as compared to routine patients at the health facility level is said to be a strong motivation for parents to allow their children to be enrolled into new drug trials. The findings showed that trial patients receive better attention, promptness of care and quality medicine. An interesting observation in the qualitative discussions is the association of packaging of drugs to trial participants where pre-parked drugs are mostly given to them. They said this makes the drug very effective. The use of water to mix and serve drugs from big containers into smaller bottles at dispensaries to routine patients is associated with weak efficacy. It is believed that the use of water to mix drugs and the process of pouring drugs into small bottles weakens the strength and effectiveness of the drug. Of course observing good hygienic practices in the dispensation of drugs would avoid contamination and ensure good quality drugs. The cost of treatment for patients involve in trial studies is lower than that of the routine patients. Most of the parents reported that their children had good health when they took part in the trial studies because of the quality of medicines they receive [2, 3].

A good relationship between researches and communities in which research studies are conducted is essential for successful conduct of trials studies. The extent to which trials are beneficial or perceived to be beneficial by participants influences the level of community cooperation and support towards the conduct of these trial studies. Irrespective of whatever benefits a trial portends, it is important that in the mind’s eye of members of the community and parents, the trial should be seen as beneficial. Almost all the parents who were part of this study said that these trials are very useful and beneficial to them and their children in so many ways.

The results established that about 79 % (310) of respondents mentioned good health for them and their children as the main benefit they get when their children took part in these trial studies. In the qualitative discussions, most parents mentioned free medical care, health education, prompt and appropriate medical attention to ensure good health for their children as the main motivation factor for their involvement in these trial studies [18]. These benefits are in line with expectations of parents prior to their children involvement in the trial studies. Our results collaborate with findings from earlier studies that reported that trials participants were given free medical care [2–4]. Free health insurance registration for study participants has also been reported one of the benefits for being involved in new drug trials [3, 11]. Our study setting is largely rural and poor but it is not clear that material benefits attract parents to allow their children to take part in these trials. What is however clear is the placement of high premium on the quality of health care received during the conduct of these trials. This is directly in line with what parents and other community members would usually expect before taking the decision to be involved in these trial studies [2, 3].

### Disease prevention

Closely related to the benefits trial studies bring to trial participants, there is the strong tendency that trial studies would help to reduce the occurrence of diseases at the community level. The qualitative findings showed that trial studies have contributed immensely in reducing disease occurrences and even help to eradicate certain diseases at the community level. Diseases like measles and convulsion have been eradicated while the rate at which people are infected by malaria, cerebrospinal meningitis, cholera, elephantiasis and diarrhoea especially in children have been reduced. This was attributed to the efforts of the NHRC. While acknowledging the important role of NHRC in health promotion in the study area, parents appear to have an exaggerated perception of its contribution with the neglect of efforts made by the formal health system in this regard. For instance, the NHRC has not played a major role in the eradication of measles yet the Centre is easily credited with such achievement. This strong perception by parents has made them to develop so much trust to the activities of the centre and this motivates them to participate fully in the activities of the research centre including the conduct of these trial studies. The results showed that about 99 % of parents said that the conduct of trial studies has helped to reduce the prevalence of malaria in the study area. It is reported that the conduct of clinical trials is effective in preventing disease like diarrhoea, preventing complications and reduced hospital attendance [1].

Purported reductions in disease occurrence, free medical care and the other benefits people get from participating in the conduct of trial studies, promotes savings for other household needs and this has affected their living standard positively hence explains why they are willing to be involved in the conduct of these new drug trial studies.

Clinical trial participants in this setting are exposed to some disadvantages or risks for their involvement in new drug trials. A particular concern is the crying of children during samples taking and likely side effects of new drugs such as burning of triode and intestines. It is demonstrated that uncertainty in the efficacy of new drugs and side effects of new drugs may have on the liver and kidney as serious risk to trial participants [3, 5, 11]. It is therefore important that clinical trials in this and similar settings ensure full disclosure of information including anticipated risks, benefits and trial procedures [3]. Most of the parents who took part in this study however, expressed strong belief and trust in the efficacy of study drugs used in this setting. Apparently, this trust is based on long history of good relationship in the conduct of research by the NHRC over the years in the communities.

## Conclusion

It is necessary to highlight the crucial role played by stakeholders and other community members in the conduct of clinical trials in this setting over the years. There are certain factors that motivate community members’ trust and willingness to offer themselves available to be use in new drug trials. These include free medical care, quality and availability of health care services, improve health status, disease prevention among others. Generally, the implementation of clinical trials in the Kassena-Nankana districts is believed to have positive impact on health status of people particularly those who participated directly in these trials.

Despite the fact that all these benefits are being enjoyed by community members as a result of the conduct of clinical trials in communities, certain issues have been raised by parents such as the safety, likely risks and side effects of new drugs. It is therefore recommended that pragmatic steps be taken by researchers to address the few concerns raised by the stakeholders on the conduct of clinical trials. This will help improve and sustain the kind of trust community members have built over the years towards the conduct of clinical trials. This will help strengthening their relationship with researchers and consequently encourage communities continue support and involvement in the conduct of clinical trial studies.
